# The Effects of a Locally Developed mHealth Intervention on Delivery and Postnatal Care Utilization; A Prospective Controlled Evaluation among Health Centres in Ethiopia

**DOI:** 10.1371/journal.pone.0158600

**Published:** 2016-07-06

**Authors:** Solomon Shiferaw, Mark Spigt, Michael Tekie, Muna Abdullah, Mesganaw Fantahun, Geert-Jan Dinant

**Affiliations:** 1 Department of Reproductive Health and Health Service Management, School of Public Health, College of Health Sciences, Addis Ababa University, Addis Ababa, Ethiopia; 2 Maastricht University, CAPHRI School for Public Health and Primary Care, Department of Family Medicine, Maastricht University, Maastricht, The Netherlands; 3 General Practice Research Unit, Department of Community Medicine, the Arctic University of Norway, Tromsø, Norway; 4 UNFPA - Liaison Office, Addis Ababa, Ethiopia; 5 UNFPA - East and Southern Africa Regional Office, Johannesburg, South Africa; Stellenbosch University, SOUTH AFRICA

## Abstract

**Background:**

Although there are studies showing that mobile phone solutions can improve health service delivery outcomes in the developed world, there is little empirical evidence that demonstrates the impact of mHealth interventions on key maternal health outcomes in low income settings.

**Methods:**

A non-randomized controlled study was conducted in the Amhara region, Ethiopia in 10 health facilities (5 intervention, 5 control) together serving around 250,000 people. Health workers in the intervention group received an android phone (3 phones per facility) loaded with an application that sends reminders for scheduled visits during antenatal care (ANC), delivery and postnatal care (PNC), and educational messages on dangers signs and common complaints during pregnancy. The intervention was developed at Addis Ababa University in Ethiopia. Primary outcomes were the percentage of women who had at least 4 ANC visits, institutional delivery and PNC visits at the health center after 12 months of implementation of the intervention.

**Findings:**

Overall 933 and 1037 women were included in the cross-sectional surveys at baseline and at follow-up respectively. In addition, the medical records of 1224 women who had at least one antenatal care visit were followed in the longitudinal study. Women who had their ANC visit in the intervention health centers were significantly more likely to deliver their baby in the same health center compared to the control group (43.1% versus 28.4%; Adjusted Odds Ratio (AOR): 1.98 (95%CI 1.53–2.55)). A significantly higher percentage of women who had ANC in the intervention group had PNC in the same health center compared to the control health centers (41.2% versus 21.1%: AOR: 2.77 (95%CI 2.12–3.61)).

**Conclusions:**

Our findings demonstrated that a locally customized mHealth application during ANC can significantly improve delivery and postnatal care service utilization possibly through positively influencing the behavior of health workers and their clients.

## Introduction

It is estimated that 287 000 maternal deaths occurred in 2010 mostly in low and middle income countries from preventable causes. [1,2] With one of the highest maternal mortality rates in the world, improving maternal health remains a major challenge for the health system in Ethiopia. A major contributory factor for the slow progress is the low level of skilled attendance at delivery (10%) and postnatal care (PNC) (7%), which in turn is influenced by the low ANC service utilization at 34%. [3,4]

Service access is generally higher in the urban areas as indicated by a relatively higher percentage of women (69%) having at least one antenatal care (ANC) visit from health professionals compared to only 24% among rural women. [4] Nevertheless, the percentage of pregnant women delivering in the presence of a skilled health professional even in urban areas is much lower than expected at 51% [4]. Previous studies show that pregnant women may not use maternity care services because of poor satisfaction, which emanates from concern about privacy and mothers’ previous negative experiences with the health system. [5,6] Other important barriers of service utilization include distance to the health facility and cost of service. [7] The fact that nearly half of mothers in urban areas do not deliver in health facilities despite better geographical access, shows the missed opportunity to encourage institutional delivery. [4]

Mobile devices have shown tremendous promise to bridge the digital gap in developing countries, with increasing penetration (unique subscriber penetration rate of 38% in 2014 for sub-Saharan Africa), better functionality and lowering of prices. [8] There is growing evidence showing that commonly employed mobile phone solutions (mHealth) such as text messaging (SMS), video messaging, voice calling, and internet connectivity can improve health service delivery processes and health outcomes, particularly in the areas of treatment adherence, appointment compliance and patient monitoring in the developed world [9–16]. Studies from Ethiopia showed mobile phones can be used by community health workers and midwife nurses for their day-to-day work and that local ownership is key for a successful mHealth program. [17, 18] Another study from Tanzania showed mobile phone intervention in the form of SMS and mobile phone voucher can significantly increase skilled delivery attendance amongst women of urban residence. [19] However, there is little evidence that demonstrates the impact of mHealth interventions on the broader list of key maternal health service outcomes, including repeat visit for antenatal care, health facility delivery and postnatal care in low income settings which made it difficult to support its implementation and scale up. [20–23]

The present study aimed to determine whether an mHealth intervention and training of health providers on client centered care, can improve maternity service utilization specifically repeat ANC attendance, institutional delivery, and PNC service utilization compared to the conventional approach. The mHealth interventions in this study included reminders of subsequent visits of ANC, delivery and PNC as well as educational messages on dangers signs during pregnancy and common complaints during pregnancy sent to health workers on a weekly basis from a central server based at Addis Ababa University.

## Methods

### Study Setting

The study was conducted in Semen Shewa Zone, Amhara region, central Ethiopia in a total of 10 health facilities (5 intervention, 5 control) serving around 250,000 people. Health facilities within 10 km of the main road connecting Addis Ababa to North East Ethiopia were included for both intervention and control arms of the study. The main purpose of limiting the study sites within the above distance was to ensure that all study participants had sufficient mobile phone network connectivity and similar physical access to health facilities. Health centers in the non-bordering districts (more than 50 km away from the nearest intervention health center) were considered as control to minimize intervention contamination (the possibility of mothers in one of the intervention groups moving to the other health centers or vice versa because of perceived improved care in the intervention areas). These health centers were also within 10 km of the main road.

### Study Design and Sampling Strategy

The study was an interventional study by design with intervention and control health facilities with comparable endpoints. The study used two approaches to gather data from women visiting the health facilities for obstetric care;

1. Cross-sectional surveys. These were exit interviews among consecutive ANC attendants in intervention and control health centers who visited the health centre before and at 12 months follow-up.
2. A longitudinal follow-up of women (from their obstetric record) who visited the selected health facilities for ANC, delivery and PNC. The obstetric record reviews were done at 12 months after implementation of the intervention, including all women who visited the health centre in the six months preceding the follow-up measurement.

**Fig 1:**
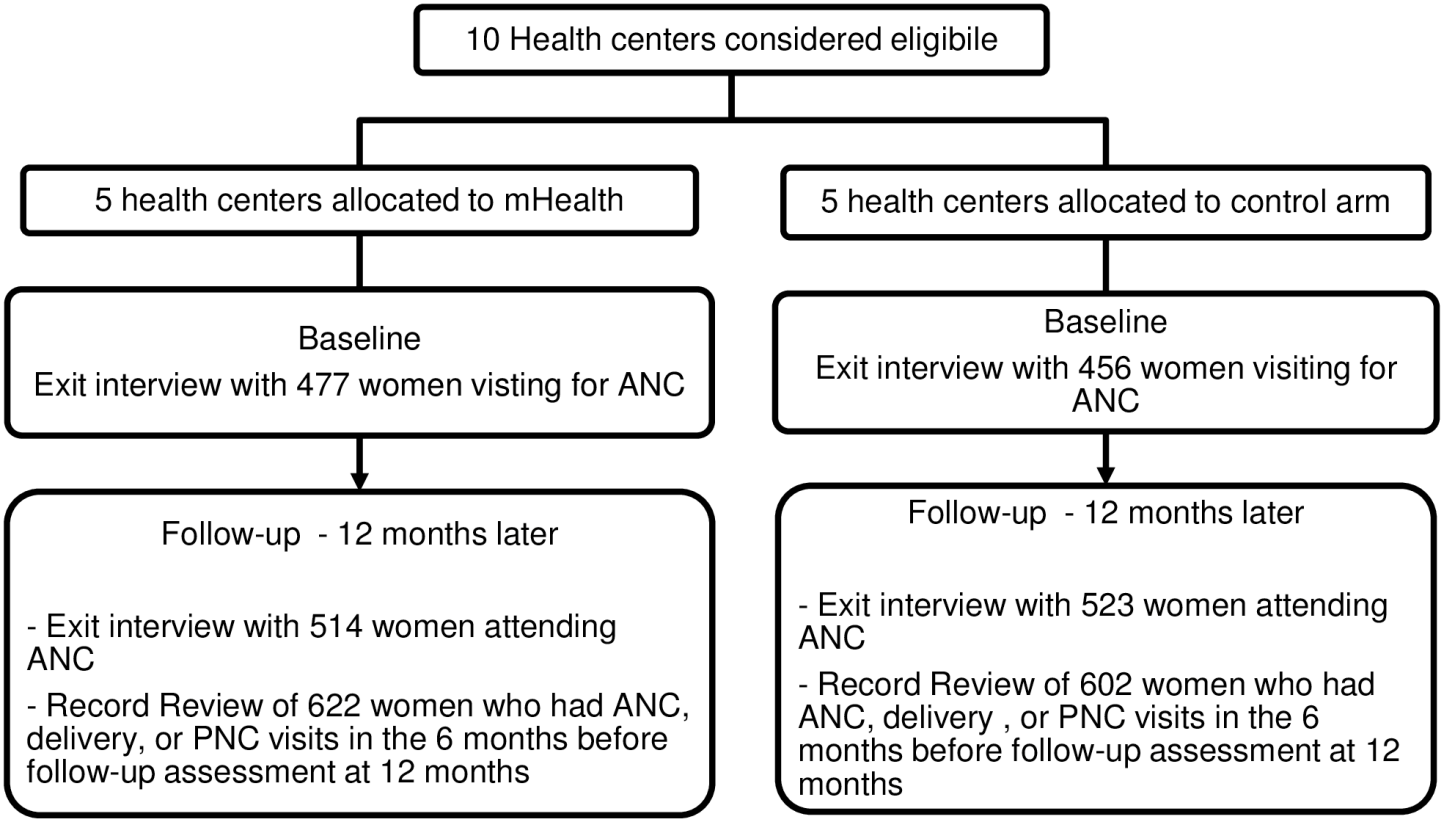
Sampling procedures and follow up schedule.

It is important to note that women followed in the longitudinal study are not necessarily the same women who participated in the cross-sectional surveys. The sampling procedure and follow-up schedule is shown in Fig 1.

### Description of the Intervention

The health workers (nurses/health officers—A nurse and health officer are health professionals with at least 2 and 4 years of training respectively and providing maternal and child health care is one their key activities. -) in the intervention group received an android phone loaded with an application that had four major features described below. First health workers were requested to register all clients who came to their health facility for ANC, delivery care or PNC using the same forms they would otherwise fill in using the paper based registry book. The application generated a unique identification code based on the names and other background information of each pregnant woman (Kebele which is comparable to ‘village’ and is the smallest administrative unit in Ethiopia where approximately 5,000 people live, House number) allowing longitudinal follow up of each pregnant woman and sending a reminder to the health worker for each follow up visit. A scanned copy of the obstetric form is shown in S1 supporting information.

### Major features of the mobile phone application

1. Reminders for subsequent visits of clients to health workers
  - Based on the information of the estimated duration of pregnancy (gestational age or month of amenorrhea), the software application calculated the next recommended ANC visits (at weeks 14, 24, 30 and 36) and the expected date of delivery at the 40^th^ week of gestational age. The reminders for the two PNC visits (on weeks 1 and 6) were based on the date of birth information for mothers who gave birth in the health center. Reminders for ANC/delivery and PNC were sent to health workers 7 days and 3 days before the scheduled visits respectively.
  - Once health workers received the reminders, they were expected to contact the client through a phone call (which could be a fixed line of neighbors, for women who did not have mobile phones). The reminder also included details about the name and phone number of the pregnant woman, so that she could be reached immediately. The project covered the cost for sending the data onto the server and calling clients which was around $10 per month for a health worker.
2. Educational messages
  - The application sent messages on danger signs during pregnancy as well as common complaints of pregnancy to health workers on a weekly basis. The messages were not specific to certain women, but were intended to refresh the knowledge of health workers on general danger signs and complaints during pregnancy. Content of these messages is shown in S2 and S3 supporting information.
3. Decision support to health workers
  - The application assisted the health worker in deciding who was eligible to receive ‘Routine ANC/Basic care’ versus ‘Specialized Care’, based on the responses given to the screening questions on the ‘Integrated Antenatal, labor, delivery, Newborn and Postnatal card’ as they were expected to complete for each mother per the Ministry of Health protocol which is shown in supplemental panel 1 [24].
4. The Report Module
  - The report included the total number of visits, round of visit (from 1^st^ to 4^th^ visit or more), and type of visit (ANC, Delivery, PNC) for each pregnant woman for any given period. The application generated this brief report on the phone, while more detailed reports could be generated through the web-based application. The report helped health workers to save the time and effort they used to spend compiling paper-based monthly reports about pregnant women served in the health facility and was developed at the request of health workers.

The project provided an air-time worth 10 US dollars per month for each health worker to cover the cost for submitting the forms and calling pregnant women ahead of their scheduled visits. Health workers were able to fill in the forms off-line and needed the cellular connection only to submit the forms and receive the reminders/educational messages and generate report on service utilization. The software application (an open-source package called Open Data Kit (ODK—available on https://opendatakit.org/)) was adapted locally by a team of IT professionals working at Addis Ababa University and was continuously modified based on the actual needs of the health workers. Complete data were transmitted to the central server at Addis Ababa University using the cellular network of the phone. The application can be shared with interested users upon request.

### Study Endpoints

The effectiveness of the intervention was evaluated by comparing the differences in the following 3 indicators in the intervention and control health centers after 12 months of implementation of the intervention;

- percentage of women who had at least 4 ANC visits
- percentage of women with Institutional delivery
- percentage of women who had the first PNC visit within 6 hours of delivery

Information regarding the 3 outcome measures was obtained through a facility record review using a standard checklist. To achieve the required sample size maternity service utilization data for 6 months (July to December 2014) were compared. Intervention started in February 2014.

### Sample Size

Sample size was determined based on the following assumptions; Proportion of pregnant women who had institutional delivery at birth among those who had at least one ANC visit—p1 = 21% [25] as control and taking a 10% difference in proportion between the intervention and control areas to signify public health importance (p2 = 31%); α error (two sided)– 0.05; 90% power; n2/n1 (ratio between the intervention and control areas)– 1:1. With a 15% upward adjustment for possible non-response, the sample size was computed approximately as n1 = n2 = 500.

### Training of Health Workers

A total of 15 Health workers (three per health center) received a two-day initial training and then quarterly (every 3 months) a refresher training (2 days each) on the mobile phone application, its potential use to improve data management and maternity care as well as the central role of client-centered care in ensuring quality care. The training focused on three main areas of maternity care: antenatal care (including birth preparedness and complication readiness), delivery care and postnatal care. The principal investigator and the IT team (2–3 people) gave the trainings.

### Evaluation and Follow-Up

Considering the median duration of pregnancy at first ANC visit for urban Ethiopia (at 4.4 months) [4], it was necessary to follow the cohort of pregnant women who had their first visit when the study began for at least 5–6 months to continue the observation until delivery and immediate postpartum.

The IT staff provided solutions in situations where health workers accidentally deleted electronic forms or faced technical challenges to transmit data to the central server.

### Data Analysis

First we compared the baseline and follow-up characteristics of antenatal care clients in the intervention and control health centers using X^2^ tests. We ran logistic regression models with intervention status as primary independent variable and the primary outcomes (having 4 ANC visits, Institutional delivery and PNC visit within 6 hours of delivery) as dependent variables, with age, residence and parity as co-variates.

To examine the possibility of 'dose-response' relationship, differences in the primary outcomes (Institutional delivery and PNC visit) between women who had one and four ANC visits were compared using the 95%CI for the difference of proportions. The criterion for significance was set at α = 0.05. All analyses were conducted with STATA version 12.

Respondents gave informed verbal consent after they were informed about the purpose of the study as described below. Data collectors read the information sheet and consent form which explained the objectives and benefits of the research, that their participation should be voluntary, confidentiality of information collected, their right to withdraw from the study without any consequence, and the minimum risk involved by participating in the study. Study participants were also given the contact addresses (phone numbers and email addresses) of the principal investigator and IRB chairperson in case they have questions to clarify. Once potential respondents decided to participate in the study, data collectors signed on the consent form and proceeded with the interview. The research protocol was approved by the Institutional Review Board(IRB) of the College of Health Sciences at Addis Ababa University (Protocol number 040/12/SPH). The study was financially supported by United Nations Population Fund (UNFPA) Ethiopia Country Office.

## Results

Overall 933 (477 in the intervention and 456 in the control) at baseline and 1037 women (514 in the intervention and 523 in the control) at follow-up were included in the cross-sectional surveys. In addition, the medical records of 622 (intervention) and 602 (control) women who had at least one antenatal care visit were followed in the longitudinal study. One health worker from one of the health centers left her work during the course of implementation of the intervention.

Table 1 shows the characteristics of the population as observed during the two cross sectional assessments. It showed that close to half of women who visited the intervention health centers were from urban areas both at baseline and follow-up. Significantly more urban women were represented at baseline in the control health centers (71.1% Vs 28.9%; p-value<0.001). With regard to education, a higher percentage of women in the intervention group had no formal education (47.8%) compared to those in the control group at baseline (38.4%) (p-value = 0.171). A significantly larger proportion of women visiting the control health centers reported to have owned a mobile phone (69.5% Vs 41.7%; p-value<0.001) at baseline. The distribution of study participants was similar according to residence and age in the follow-up survey.

**Table 1:** Percentage distribution of respondents by background characteristics—at Baseline and Follow-up.

Characteristic	Baseline			Follow-up		
	Intervention (n = 477)	Control (n = 456)	P-value	Intervention (n = 514)	Control (n = 523)	P-value
**Total**	**51.1**	**48.9**		**49.6**	**50.4**	
**Residence**			<0.001			0.158
Urban	52.4	71.1		45.5	49.9	
Rural	47.6	28.9		54.5	50.1	
**Age**			0.171			0.321
15–24	41.1	43.0		43.2	42.0	
25–34	46.3	48.3		46.9	46.6	
35–49	12.6	8.8		9.9	11.4	
**Parity**			0.492			0.237
Nulliparous	42.4	45.6		42.8	37.7	
1 to 2	36.9	33.3		40.5	44.6	
3 or more	20.8	21.1		16.7	17.8	
**Educational Status**			0.001			0.030
No formal education	47.8	38.4		33.1	40.3	
Primary (1–8)	32.7	45.8		44.4	43.2	
Secondary (9–12)	11.1	11.8		17.1	12.1	
Higher (12+)	8.4	3.9		5.5	4.4	
**Months pregnant at first visit**			<0.001			<0.001
1–3 months	31.2	45.6		37.7	47.8	
4–6 months	50.3	44.9		49.2	44.0	
7–9 months	18.0	9.4		8.8	8.0	
Don’t know	0.4	0.0		4.3	0.2	
**Number of pregnancy visits**			0.008			0.375
One	40.5	31.4		32.7	29.1	
Two	32.7	32.7		25.3	29.8	
Three	19.1	24.8		23.5	22.8	
Four & above	7.8	11.2		18.5	18.4	
**Own a mobile phone**			<0.001			0.784
Yes	41.7	69.5		49.4	48.6	
No	58.3	30.5		50.6	51.4	

Among women who sought care in the 6 months preceding the follow-up survey, as observed in the longitudinal study, the median gestational age at first ANC visit was 22.5 weeks and 22.0 weeks in the intervention and control health centers respectively (data not shown). In contrast, the median gestational age at the fourth ANC visit was 37 weeks and 36 weeks in the intervention and control group respectively (p-value<0.001). There was no statistically significant difference according to parity, and marital status (data not shown).

### Effect of the Intervention on Having at Least Four ANC Visits

As shown in Table 2, the longitudinal study showed that women in the intervention health centers were more likely to have at least 4 antenatal visits (27.0% versus 23.4%; AOR: 1.31(95%CI 1.00–1.72)) although it was not statistically significant. Women from urban areas were significantly more likely to have at least four antenatal care visits compared to rural living women (26.9% versus 20.9%: AOR: 0.68(95%CI 0.50–0.93)). There was no statistically significant difference by age, and parity.

**Table 2:** Determinants of having at least four Antenatal Care visits in a health center.

	Four ANC visits					
Characteristics	Yes		No		OR	Adjusted OR
	n	%	n	%		
Study Arm						
mHealth Intervention	168	27.0	454	73.0	1.21 (0.93, 1.57)	1.31 (1.00, 1.72)
Control	141	23.4	461	76.6	1.00	1.00
Age						
15–24yrs	140	26.1	396	73.9	1.00	1.00
25–34yrs	132	24.5	406	75.5	0.91 (0.70, 1.21)	0.98 (0.71, 1.35)
35–50yrs	30	23.4	98	76.6	0.87 (0.55, 1.36)	1.03 (0.59, 1.78)
Residence						
Urban	231	26.9	627	73.1	1.00	1.00
Rural	76	20.9	288	79.1	0.71 (0.53, 0.96)	0.68 (0.50, 0.93)
Parity						
None	128	27.4	340	72.7	1.00	1.00
1 to 2	117	26.1	331	73.9	0.94 (0.70, 1.26)	0.99 (0.72, 1.36)
3 or more	63	21.9	225	78.1	0.74 (.53, 1.05)	0.81 (0.52, 1.25)
**Total**	**309**	**25.3**	**915**	**74.8**		

### Effect of Intervention on Delivery in a Health Center

The multivariate analysis showed that women who visited the intervention health centers were significantly more likely to have delivery in the health center compared to the control group (43.1% versus 28.4%; AOR; 1.98 (95%CI 1.53–2.55)). See Table 3 for details.

**Table 3:** Distribution of women who delivered in the Health Center by Intervention status/characteristics.

	Delivery in HC					
Characteristics	Yes		No		OR	Adjusted OR
	n	%	n	%		
Study Arm						
mHealth Intervention	264	43.1	349	56.9	1.63 (1.29, 2.06)	1.98 (1.53, 2.55)
Control	161	28.4	407	71.7	1.00	1.00
Age						
15–24yrs	193	37.0	328	63.0	1.00	1.00
25–34yrs	183	35.1	339	64.9	0.93 (0.72, 1.18)	1.00 (0.75, 1.36)
35–50yrs	40	33.9	78	66.1	1.01 (0.68, 1.50)	1.01 (0.60, 1.70)
Residence						
Urban	291	35.3	533	64.7	1.00	1.00
Rural	133	37.5	222	62.5	1.04 (0.82, 1.35)	1.04 (0.79, 1.36)
Parity						
None	182	39.8	275	60.2	1.00	1.00
1 to 2	158	36.4	276	63.6	0.89 (0.68, 1.16)	0.89 (0.66, 1.19)
3 or more	84	30.8	189	69.2	0.74 (0.55, 1.01)	0.70 (0.46, 1.05)
Frequency of ANC visits						
One	101	22.5	348	77.5	1.00	
Two	84	34.3	161	65.7	1.75 (1.26, 2.44)	
Three	94	42.9	125	57.1	2.54 (1.82, 3.55)	
Four	145	54.5	121	45.5	3.74 (2.72, 5.15)	
**Total**	**425**	**36.0**	**756**	**64.0**		

### Effect of Intervention on PNC

As shown in Table 4, a significantly higher percentage of women in the intervention group had PNC in the health centers compared to the control health centers (41.2% versus 21.1%: AOR; 2.77 (95%CI 2.12–3.61).

**Table 4:** Results from a logistic regression analysis of determinants of Postnatal care in a Health Center.

	PNC in HC					
Characteristics	Yes		No		OR	Adjusted OR
	n	%	n	%		
Study Arm						
mHealth Intervention	252	41.2	360	58.8	2.51 (1.96, 3.20)	2.77 (2.12, 3.61)
Control	124	21.1	465	79.0	1.00	1.00
Age						
15–24yrs	166	31.4	362	68.6	1.00	1.00
25–34yrs	162	30.7	366	69.3	0.92 (0.72, 1.19)	1.03 (0.76, 1.41)
35–50yrs	40	32.5	83	67.5	1.00 (0.66, 1.49)	1.11 (0.65, 1.87)
Residence						
Urban	250	29.7	593	70.3	1.00	1.00
Rural	125	35.1	231	64.9	1.30 (1.01, 1.67)	1.16 (0.88, 1.54)
Parity						
None	161	34.8	302	65.2	1.00	1.00
1 to 2	136	30.8	306	69.2	0.86 (0.67, 1.13)	0.83 (0.61, 1.14)
3 or more	78	28.0	201	72.0	0.74 (0.54, 1.01)	0.68 (0.45, 1.04)
Frequency of ANC visits						
One	88	19.6	362	80.4	1.00	
Two	73	29.2	177	70.8	1.56 (1.12, 2.19)	
Three	83	36.2	146	63.8	2.00 (1.42, 2.80)	
Four	131	48.5	139	51.5	3.28 (2.38, 4.51)	
**Total**	**376**	**31.3**	**825**	**68.7**		

### Effect of Prolonged Exposure to the mHealth Intervention on Delivery and PNC

**Fig 2:**
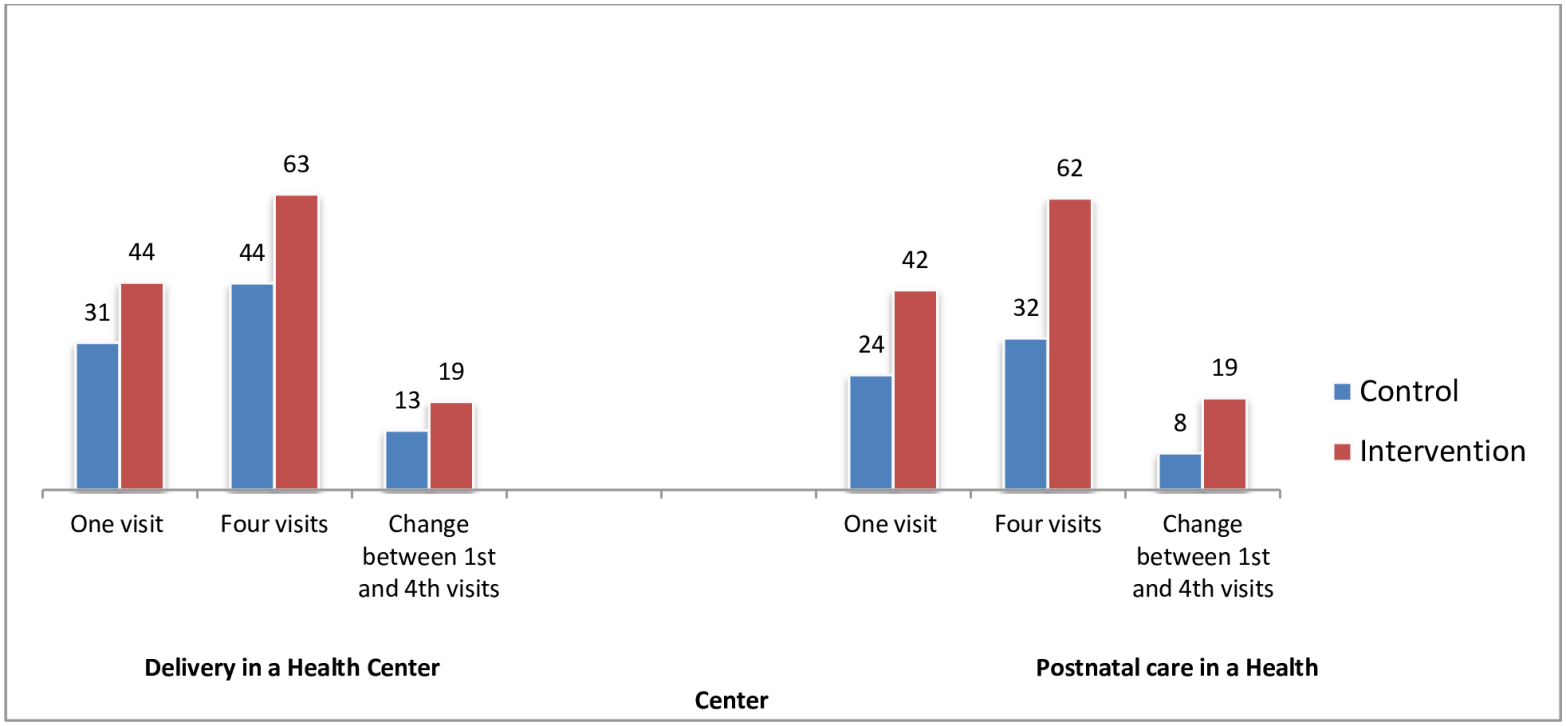
Percentage distribution of women who had Delivery and Postnatal care in a Health Center according to number of ANC visits.

Fig 2 reports the significant increase in percentage of women who had delivery and postnatal care in the health center as the number of visits increase. The difference in the percentage of delivery between women with one and four antenatal care visits in the intervention health centers was 18.7 percentage points (44.0% versus 62.7%, p-value<0.001) compared to the control health centers 12.6 percentage points (31.2% versus 43.8%, p-value = 0.004). Likewise, the difference in the percentage of women having PNC among women with one versus four antenatal care visits in the intervention and control was 19.7% (43.3% versus 63.0%, p-value<0.001) and 8.2% (24.7% versus 32.8%, p-value = 0.063) respectively.

## Discussion

In this study we found that using a phone based application that has features of decision support, sending reminders about subsequent visits and educational messages, significantly increased delivery and postnatal care service utilization regardless of age of women, residence (urban/rural) and parity. The percentage of women having institutional delivery and postnatal care increased by close to 15 and 20 percentage points respectively in the intervention group compared to the control.

It is likely that the intervention effect is mediated through several mechanisms. First, it appears that the reminders encouraged health workers in calling pregnant women to come on their appointment days and hence have more frequent antenatal care visits. Having four antenatal visits was one of the intermediate outcomes that was associated with higher odds of institutional delivery and postnatal care. Secondly, having contact numbers of their clients (or neighbors for those who do not have personal mobile phone), helped health workers to track and respond to the health needs of pregnant women in a more efficient way. It is also possible that the very act of asking phone numbers contributed to clients feeling more valued by health workers and hence be responsive to the advice they received at health facilities. Third, the weekly educational messages on danger signs and common complains might have helped boost the knowledge of some health workers on what signs to look for and provide appropriate counseling and referral.

Recent systematic reviews show lack of studies with appropriate design that document the effectiveness and cost-effectiveness of phone-based applications on objective maternal health outcomes as well as chronic diseases in low income country settings [20,26–28]. Although it is difficult to draw direct comparison due to differences in study designs, type of mobile application and target audience, our findings are generally consistent with previous studies in the developed world and some low-income countries that demonstrated that reminders can improve skilled birth attendance at delivery [19] and adherence to anti-retroviral treatment and smoking cessation [26,29].

Further, our study showed that nearly half of antenatal care attendants in the study area have some form of mobile phones which is indicative of the huge opportunity in creating synergy by targeting clients with relevant reminders and educational messages. Currently, there is a global movement to preload generic health related messages in local languages at the time of manufacturing to empower the population with accurate information [30]. At a time the phone manufacturing sector is growing fast, it is important to start the discussion on how best to encourage production of phones with preloaded messages [31]. This needs to be supplemented by development of more specific educational messages (both to health workers and clients) depending on women's gestational age and pre-existing medical conditions.

### Strength and Limitations

There are a couple of limitations of the present study that merit attention. The fact that there was limited background information about women in the medical records restricted our ability to account for all possible confounders in the final regression analysis. However, the survey at baseline showed a higher proportion of women in the control group had better education and mobile phone ownership. Considering that one would expect a better outcome in higher educated women and in women who own a mobile phone, it is more likely that we underestimate the magnitude of the effect of our intervention, if at all such bias exist.

The fact that the application was designed in Ethiopia using only local resources, and an open software platform, which is available freely to everyone is one of the features of this intervention that helped in continuous improvement and utilization by the health workers and health officials responsible for managing the facilities. We also believe this is one of the few studies which was conducted with strong study design addressing important maternal health outcomes in a low-income setting.

## Conclusions and Implication for Practice

Our study has demonstrated that the mHealth intervention package(in the form of decision support, reminders, educational messages and report) had positive impact on delivery and postnatal care in health facilities regardless of residence, age and parity of women possibly through positively influencing the behavior of health workers and their clients.

The study showed that the benefit in terms of ensuring adherence to the recommended visits does not extend beyond the immediate postpartum period showing the need to convince clients of the added value of a repeat visit at 6 weeks postpartum. There is also a need to identify determinants of postnatal service utilization at 6 weeks and beyond so that the whole continuum of maternity care can be effectively addressed.

One of the key motivating factors for the health workers to use the application was the fact that the application made it easier for them to report on the number of pregnant women with their respective number of visits. This helped them to avoid the manual work they had to do previously to compile the information, and it also showed them the level of adherence of their clients to the scheduled visits. One of the key lessons we learned in the implementation process was to be responsive to the immediate demands of health workers as much as possible to ensure proper utilization of the application.

We also believe active participation of health workers in the whole process with flexibility to address real challenges in the health system helped to ensure local ownership and contributed to the success of mHealth interventions.

Future studies need to explore the possible mechanisms of action of various components of existing mobile health solutions and evaluate their effectiveness in other priority health programs such as malnutrition, family planning and immunization of children on a bigger scale. Finally, although we have-not conducted a cost-effectiveness analysis, it is possible to further decrease the cost of implementation of such projects by providing more applications that offer practical solutions to health challenges that are likely to benefit from technology based innovations.

## Supporting Information

S1 Supporting Information Integrated Antenatal, Labor, Delivery, Newborn and Postnatal care card. (DOCX)

S2 Supporting Information Danger Signs During Pregnancy. (DOCX)

S3 Supporting Information Common complaints during pregnancy. (DOCX)
