# Supplementary material for: The Effects of a Locally Developed mHealth Intervention on Delivery and Postnatal Care Utilization; A Prospective Controlled Evaluation among Health Centres in Ethiopia
Source: PLoS One. 2016 Jul 6;11(7):e0158600. doi: 10.1371/journal.pone.0158600 (PMC4934867; doi:10.1371/journal.pone.0158600)
Supplement: S1 Supporting Information — (DOCX) [file pone.0158600.s001.docx]

**S1 - Integrated Antenatal, Labor, Delivery, Newborn and Postnatal care card**
