# Supplementary material for: The Effects of a Locally Developed mHealth Intervention on Delivery and Postnatal Care Utilization; A Prospective Controlled Evaluation among Health Centres in Ethiopia
Source: PLoS One. 2016 Jul 6;11(7):e0158600. doi: 10.1371/journal.pone.0158600 (PMC4934867; doi:10.1371/journal.pone.0158600)
Supplement: S2 Supporting Information — (DOCX) [file pone.0158600.s002.docx]

| **S2 - Danger Signs During Pregnancy** |
| --- |
| Teach the pregnant woman and her family to report any of the following conditions immediately: |
| - Vaginal bleeding |
| - Sudden gush of fluid or leaking of fluid from vagina |
| - Severe headache not relieved by simple analgesics |
| - Dizziness and blurring of vision |
| - Sustained vomiting |
| - Swelling (hands, face, etc.) |
| - Loss of fetal movements |
| - Convulsions |
| - Premature onset of contractions (before 37 weeks) |
| - Severe or unusual abdominal pain |
| - Chills or fever |
