# Supplementary material for: The Effects of a Locally Developed mHealth Intervention on Delivery and Postnatal Care Utilization; A Prospective Controlled Evaluation among Health Centres in Ethiopia
Source: PLoS One. 2016 Jul 6;11(7):e0158600. doi: 10.1371/journal.pone.0158600 (PMC4934867; doi:10.1371/journal.pone.0158600)
Supplement: S3 Supporting Information — (DOCX) [file pone.0158600.s003.docx]

| **S3 Common complaints during pregnancy** | | |
| --- | --- | --- |
| **Complaint** | **What to Tell the Client** | **Provider Management** |
| Constipation | • Increase your water intake (8 glasses); eat high-fiber foods, and take daily exercise.  • Use mild laxatives as a last resort. | • Counsel the client on diet.  • Suggest mild laxatives only if the other measures have failed. |
| Headache | • Take mild pain relievers; *e.g.*, paracetamol.  • Avoid aspirin.  • Inform provider if pain becomes severe she should attend the health facility | • Determine that the headache is not a *Danger Sign* (see below).  • Offer paracetamol (Panadol) 300 mg every 3-4 hours.  • For severe headache or migraine, offer codeine or other related narcotic might be used.  ***Remember***: headache can be associated with hypertension. |
| Backache | • Avoid excessive bending, lifting, or walking without a rest period.  • Rock pelvic periodically during the day for relief.  • Wear supportive, low-heeled shoes.  • If severe, wear a maternity girdle for additional support.  • Heat or ice to back for relief, whichever is more comforting. | • Counsel regarding comfort measures.  ***Remember***: the symptoms of UTI and onset of labor include backache. |
| Nausea and vomiting | • Eat small, meals frequently. Keep crackers at bedside and eat before getting out of bed. Eat fruit or drink fruit juice before going to sleep.  • Avoid oily, spicy foods.  • Get out of bed slowly.  • Symptoms should not extend beyond the first three months; if severe and persistent, see your health care provider. | • Counsel about comfort measures.  • Provide Vitamin B6, 50 mg, twice daily.  • If symptoms are severe, refer for possible hospitalization and intravenous fluids. Medications for management may include: promethazine (Phenergan) -Diphenhydramine (Benadryl) -Other antihistamines  • Birth defects have not been associated with the use of these drugs. |
| Varicosities | • Elevate legs periodically during the day.  • Wear support hose (elevate legs before putting on hose for maximum support). | • Prescribe support hose, as necessary.  • Refer if varicosities are severe and painful. |
| Vaginal discharge | • Cleanse genitalia daily. Wear cotton underwear.  • Use light sanitary pads if discharge is heavy.  • Avoid vaginal douching.  • If discharge develops with itching, irritation or unpleasant odor, see the provider as soon as possible for treatment. | • If not infection, counsel for genital hygiene.  • With symptoms of infection, treat according to guidelines or refer for treatment. |
| Leg cramps | • During cramping, straighten leg slowly with the heel pointing and the toes upward or push the heel of the foot against the footboard of the bed or floor, if standing. • Exercise daily to enhance circulation. • Elevate legs periodically throughout the day. • Take calcium tablets daily. Eat calcium rich foods such as dairy and dark green leafy vegetables. | • Prescribe calcium carbonate or calcium lactate tablets. |
